# Out-of-Hospital Cardiac Arrest Outcomes After Ventricular Fibrillation

**DOI:** 10.7759/cureus.69291

**Published:** 2024-09-12

**Authors:** Shiva Sajja, Nofel Iftikhar, Latha Ganti, Anjali K Banerjee, Paul R Banerjee

**Affiliations:** 1 Biology, The Walker School, Marietta, USA; 2 Biology, University of Florida, Gainesville, USA; 3 Research, Orlando College of Osteopathic Medicine, Winter Garden, USA; 4 Medical Science, The Warren Alpert Medical School of Brown University, Providence, USA; 5 Emergency Medicine & Neurology, University of Central Florida, Orlando, USA; 6 Neuroscience, University of Georgia, Athens, USA; 7 Emergency Medicine, Lakeland Regional Medical Center, Lakeland, USA

**Keywords:** cardiac arrest, cardiac resuscitation, emergency medical services (ems), out of hospital cardiac arrest, ventricular fibrillation arrest

## Abstract

Introduction: This study is a retrospective review of patients who sustained out-of-hospital cardiac arrest due to ventricular fibrillation. The data were analyzed to decipher predictors of good outcomes as the overall survival rate in the county is significantly higher than the national average.

Methods: The inclusion criteria for the study comprised all patients over the age of 18 for whom a call was made for unresponsiveness. Data for this project included all cardiac arrests due to ventricular fibrillation in the calendar year 2022.

Results: A total of 80 patients sustained cardiac arrest due to ventricular fibrillation. The age range was 27-80 years old. The study has 71% White, 19% African American, 8.7% Hispanic, and 1% other populations. Ninety-five percent received epinephrine, 89% utilized an advanced airway, 60% underwent hypothermia protocol, 24% utilized an AED device, and 14% used a mechanical CPR device. Seventy-six percent were pronounced dead in the ER or the hospital, and 19% survived to discharge. In the survivor population, CPR was initiated in 13 minutes or less and defibrillation occurred in 23 minutes or less. While none of the variables achieved statistical significance, epinephrine use showed a trend toward statistical significance for the outcome of sustained return of spontaneous circulation (ROSC) with a p-value of 0.05346.

Conclusion: Nineteen percent of patients survived out-of-hospital cardiac arrests in the Polk County hospital system. This is significantly higher than the national average. This likely reflects the emphasis on high-quality CPR and active on-scene management, as no individual variable was statistically significant.

## Introduction

There are nearly 400,000 out-of-hospital cardiac arrests in North America every year, defined as any cardiac arrests that occur in non-healthcare settings [1-3]. These events have a worrying prognosis as only 10% survive out-of-hospital cardiac arrests. Without any immediate intervention, such as cardiopulmonary resuscitation (CPR), survival is impossible [3]. Following out-of-hospital cardiac arrest events, approximately 100,000 of these patients present to the ED with ventricular fibrillation (VF) or pulseless ventricular tachycardia (VT) [4]. VF is defined as a life-threatening heart arrhythmia that results from the quiver of the heart’s ventricles, instead of proper blood pumping [5,6]. VF occurs as a result of disorganized or disrupted electrical signals, which cause uncoordinated pumping of the heart's lower chambers [7]. VF represents a dangerous and life-threatening condition that presents with symptoms, including but not limited to fatigue, fainting, or an irregular heartbeat [8]. VF is a hazardous condition that can result in cardiac arrest and death, with approximately 60% of cardiovascular deaths resulting from VF [6]. VT is defined as a life-threatening cardiac arrhythmia in which rapid contractions occur, leading to heart failure [9]. Symptoms of VT include chest pain, palpitations, dyspnea, light-headedness, and syncope [10]. If untreated, VT can lead to organ failure, heart failure, and sudden cardiac arrest [10,11]. Traditionally, patients are treated for VF or VT with CPR and standard defibrillation (using 200J-300J-360J) [5,6]. If VF or VT is left untreated, refractory VF (RVF), the condition when, following three different defibrillation attempts, spontaneous circulation does not return, may develop [12-13]. Achievement of the return of spontaneous circulation (ROSC) and sustained ROSC serve as critical indications of survival possibility [14,15]. While CPR and other resuscitation measures are of paramount importance, other factors, including treatment measures utilized and patient identity descriptors, may play critical roles.

## Materials and methods

Polk County Fire Rescue (PCFR) is one of the largest EMS departments in the state of Florida, responding to more than 115,000 calls per year and covering an area of over 2010 square miles. PCFR participates in the Cardiac Arrest Registry to Enhance Survival (CARES) data registry. CARES is a secure, web-based data management system in which participating agencies enter local data and generate their own reports. PCFR has a robust research and quality program that collects data on calls and transports. The PCFR research and quality registry has exempt approval from the University of Central Florida Institutional Review Board.

Inclusion criteria for the study comprised all patients over the age of 18 for whom a call was made for unresponsiveness. Data for this project included all cardiac arrests due to VF in the calendar year 2022.

Sustained ROSC was defined as the return of spontaneous circulation maintained through the end of EMS resuscitation (arrival to the hospital). Variables for the multivariable model included administration of epinephrine, time from EMS dispatch call to arrival on the scene, if the arrest was witnessed if the patient was defibrillated, if hypothermia care was provided in the field, location of cardiac arrest (home, nursing home, outdoors), age of the patient, and sex of the patient.

Statistical analysis was performed using JMP 16.0 for Mac. Descriptive analyses are reported for demographics. Univariate associations and multivariate logistic regression modeling were performed for associations with ROSC and sustained ROSC.

## Results

A total of 80 patients sustained cardiac arrest due to VF in the study period (January 1, 2022 to December 31, 2022). The median age of the study population was 63, with an interquartile range of 52-73 and a range of 27-89.

The study population was 71% White (n=57), 19% Black (n=15), 8.8% Hispanic (n=7), and 1% (n=1) labeled as “other.” Regarding common comorbidities in the study population: 34% (n=27) had chronic hypertension, 24% had heart disease (n=19), 5% (n=4) were diagnosed with cancer, 4% (n=3) had diabetes, and 71% (n=57)had other unspecified pre-existing or chronic conditions. Concerning the location of cardiac arrest, 70% (n=57) of the cardiac arrests occurred at home, 20% (n=16) occurred outside, and 10% (n=8) occurred at a nursing home/assisted living facility. Additionally, 73% (n=58) of the arrests were witnessed, while 27% (n=22) were unwitnessed.

With respect to treatment administered, 95% of patients were given epinephrine, 89% of patients received an advanced airway, 60% of patients underwent the hypothermia protocol, an AED was used in 24%, and 14% of patients had a mechanical CPR device used on them. Emergency medical service (EMS) arrived at the scene and started CPR in under 45 minutes, defibrillation within 67 minutes, with 40% of patients achieving, at least momentarily, ROSC.

Following arrival at a hospital, 76% of the patients were pronounced dead in the ED or died during hospitalization, and 19% of the patients were discharged alive from the hospital. Out of the survivor population, CPR was initiated in 13 minutes or less, and defibrillation occurred in 23 minutes or less. Concerning survivor demographic majorities, 87% of the survivors were white, 67% of the survivors were male, 80% of the survivors had a witnessed arrest, and 73% of the survivors had their arrest occur at home.

In a multivariate model that included epinephrine, time from EMS dispatch call to arrival on scene, if the patient was defibrillated, if hypothermia care was provided in the field, location of cardiac arrest, and age and sex of the patient, only epinephrine use showed a trend toward statistical significance for the outcome of sustained ROSC, with a p-value of 0.0535. Conversely, no other variables showed a significant p-value. Although epinephrine administration was the only statistically significant variable in the overall analysis, modeling epinephrine administration against sustained ROSC in a linear regression yielded a relatively low R^2^ value of 9.4%, suggesting that there were likely other factors, such as perhaps the patient’s baseline comorbidities, that contributed to their outcome. The calculated p-values for all variables are summarized in Table 1.

**Table 1:** Predictors of ROSC and their statistical significance

Variable	P-value
Was epinephrine given?	0.05346
Time from EMS dispatch to arrival on the scene	0.12605
Defibrillation performed	0.23401
Hypothermia care provided in the field?	0.57166
Age	0.58818
Location arrest occurred	0.65503
Sex	0.95365

## Discussion

**Figure 1:**
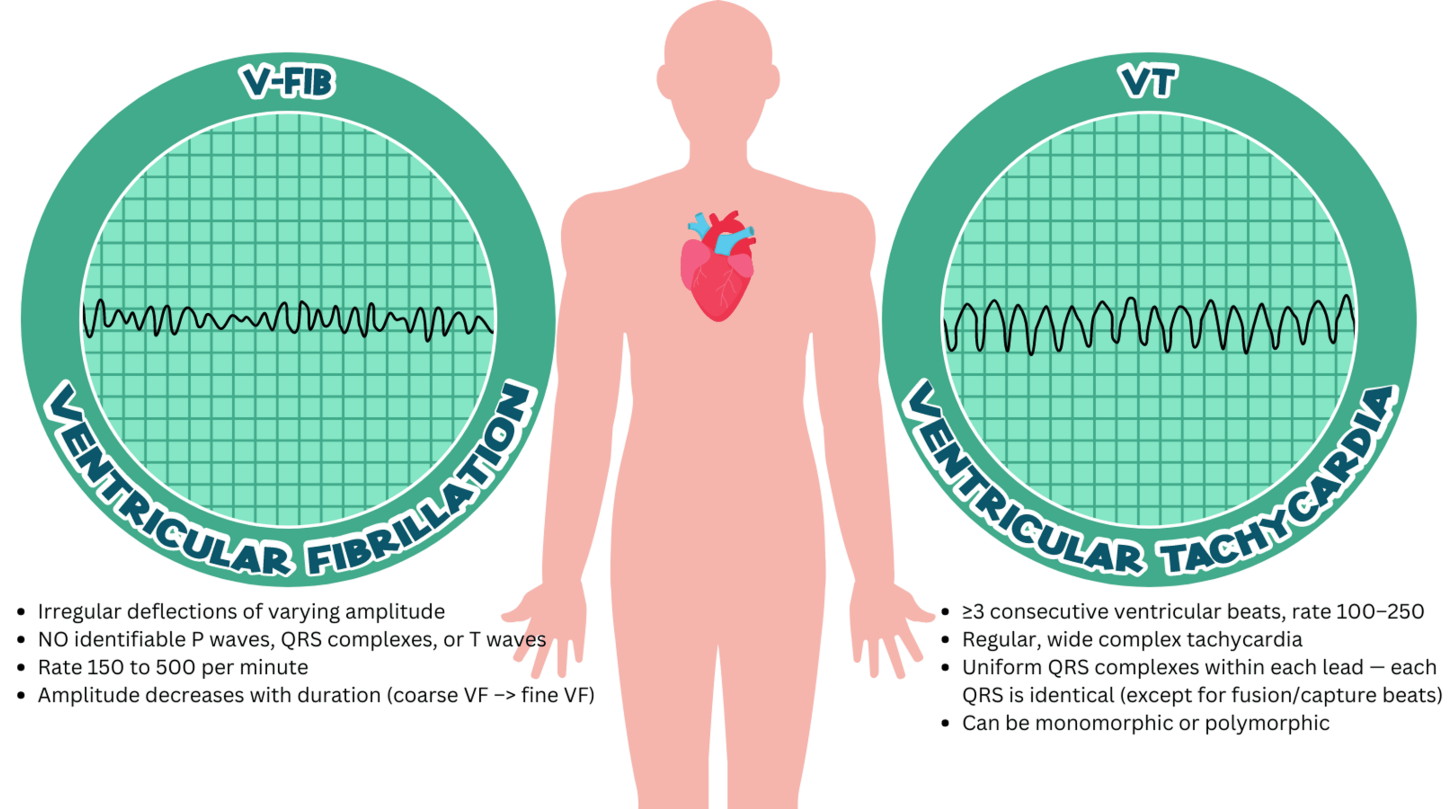
Ventricular fibrillation vs. ventricular tachycardia Infographic designed by Nofel Iftikhar on Canva.com

In this study, 80 patients met the criteria for VF in the out-of-hospital setting (Figure 1).

The majority of the patients who underwent cardiac arrest were males. Although this could be a result of several factors, the most prominent is males’ heightened tendency to heart disease when compared to females [16,17]. Women, in comparison to men, have higher concentrations of estrogen and progesterone, which aid in maintaining blood vessel health, thus decreasing the risk of heart disease and cardiovascular disease in general [18].

Additionally, most of the survivors within the dataset were White people. This can potentially be attributed to differences in economic, housing, and healthcare access/status between White populations and other minority populations, although these factors were not explicitly studied in this research. Compared to White populations, African American populations have lesser access to healthcare in the United States because of disproportion rates of economic instability, healthcare access inequality, and disparities in health literacy and health education [19,20]. In conjunction with relevant social factors, African Americans are more prone to heart disease than White populations, a fact which can be further exacerbated by common comorbid conditions in African American populations, such as chronic kidney disease, sickle cell anemia, and hypertension [21,22].

The data also demonstrated that the longer it took for defibrillation and CPR efforts to be performed on a patient, the lower their overall chance at survival became. The data also showed that EMS arriving on-site at an earlier time and starting CPR with defibrillation resulted in improved survival outcomes regarding out-of-hospital VF. Also of note is that compared to the national average of 10%, Polk County out-of-hospital cardiac arrest patients survived a staggering 19% of the time [3]. Polk County Fire Rescue places a tremendous amount of effort on the quality of CPR training and performance, including correct rate, depth, and timely ventilation, a practice that has resulted in ROSC rates ten times the national average in pediatric cardiac arrest [23,24]. Accordingly, Polk County practices regarding CPR training, out-of-hospital cardiac arrest protocols, and other EMS standards should be analyzed and implemented on a nationwide scale in an effort to improve patient care outcomes.

## Conclusions

Nineteen percent of patients survived out-of-hospital cardiac arrests in the Polk County Fire Rescue system, a statistic significantly higher than the national average. The survival chance for an out-of-hospital VF patient is determined by several factors, including but not limited to time from arrest to defibrillation (most important), patient factors, and EMS treatment. In this cohort of 80 patients, no one specific variable was found to be statistically significant with regard to the outcomes of ROSC or sustained ROSC. This suggests that the emphasis on high-quality CPR and active on-scene management likely plays a key role.
